# The clinical case for proton beam therapy

**DOI:** 10.1186/1748-717X-7-174

**Published:** 2012-10-22

**Authors:** Robert L Foote, Scott L Stafford, Ivy A Petersen, Jose S Pulido, Michelle J Clarke, Steven E Schild, Yolanda I Garces, Kenneth R Olivier, Robert C Miller, Michael G Haddock, Elizabeth Yan, Nadia N Laack, Carola A S Arndt, Steven J Buskirk, Vickie L Miller, Christopher R Brent, Jon J Kruse, Gary A Ezzell, Michael G Herman, Leonard L Gunderson, Charles Erlichman, Robert B Diasio

**Affiliations:** 1 Department of Radiation Oncology, Mayo Clinic, 200 First St SW, Rochester, MN, 55905, Minnesota; 2 Department of Ophthalmology, Mayo Clinic, 200 First St SW, Rochester, MN, 55905, Minnesota; 3 Department of Neurologic Surgery, Mayo Clinic, 200 First St SW, Rochester, MN, 55905, Minnesota; 4 Divisions of Pediatric Hematology and Oncology and Orthopedic Oncology, Mayo Clinic, 200 First St SW, Rochester, MN, 55905, Minnesota; 5 Division of Medical Physics, Mayo Clinic, 200 First St SW, Rochester, MN, 55905, Minnesota; 6 Division of Medical Oncology, Mayo Clinic, 200 First St SW, Rochester, MN, 55905, Minnesota; 7 Division of Oncology Research, Mayo Clinic, 200 First St SW, Rochester, MN, 55905, Minnesota; 8 Department of Molecular Pharmacology and Experimental Therapeutics, Mayo Clinic, Rochester, Minnesota; 9 Department of Radiation Oncology, Mayo Clinic Hospital, Phoenix, Arizona; 10 Department of Radiation Oncology, Mayo Clinic, Jacksonville, Florida; 11 Department of Radiation Oncology, Mayo Clinic, Scottsdale, Arizona

**Keywords:** Clinical review, Neoplasms, Proton beam therapy, X-ray therapy

## Abstract

**Abstract:**

Over the past 20 years, several proton beam treatment programs have been implemented throughout the United States. Increasingly, the number of new programs under development is growing. Proton beam therapy has the potential for improving tumor control and survival through dose escalation. It also has potential for reducing harm to normal organs through dose reduction. However, proton beam therapy is more costly than conventional x-ray therapy. This increased cost may be offset by improved function, improved quality of life, and reduced costs related to treating the late effects of therapy. Clinical research opportunities are abundant to determine which patients will gain the most benefit from proton beam therapy. We review the clinical case for proton beam therapy.

**Summary sentence:**

Proton beam therapy is a technically advanced and promising form of radiation therapy.

## Background

X-rays have been used to treat cancer since 1895. Advances in x-ray therapy over the years include development of linear accelerators that produce high-energy x-rays for deeper penetration. Blocking techniques were developed to contour the beam to conform to the size and shape of the tumor target. Multiple beams and angles are used to adapt the dose to the tumor and to reduce the dose to healthy organs. Advances in imaging have allowed for improved tumor delineation. Four-dimensional imaging allows measurement of motion of both tumor and normal structures during treatment. Changes in tumor size and shape during treatment can be corrected for use of adaptive radiotherapy techniques. Faster and more powerful computers allow for more accurate dose calculations and the delivery of intensity modulated x-ray beams and volumetric arc therapy. Improved patient and organ immobilization devices, along with imaging during treatment to detect patient, organ, and tumor motion (image-guided radiotherapy), enhance the accuracy of treatment delivery.

These advances were accomplished without performing conventional prospective clinical trials. The administration of concurrent radiation-sensitizing chemotherapy and biologically targeted agents has been found to improve both disease control and survival for many cancer patients.

The likelihood of tumor control through radiation therapy is related to the dose delivered to the tumor, and the likelihood of severe organ injury is related to dose to the organ and volume of the organ exposed to radiation [1]. A balance always exists between cure and risk of severe complications. The challenge in using high-energy x-rays to treat cancer is that the x-rays pass through the thickness of the body, depositing an entrance and an exit dose to healthy organs. The dose to healthy organs limits the dose that can be safely administered to the tumor. Radiation oncologists constantly strive to find the optimal balance between a high-enough dose to prevent cancer recurrence and a low-enough dose to avoid injury to healthy organs.

**Figure 1:**
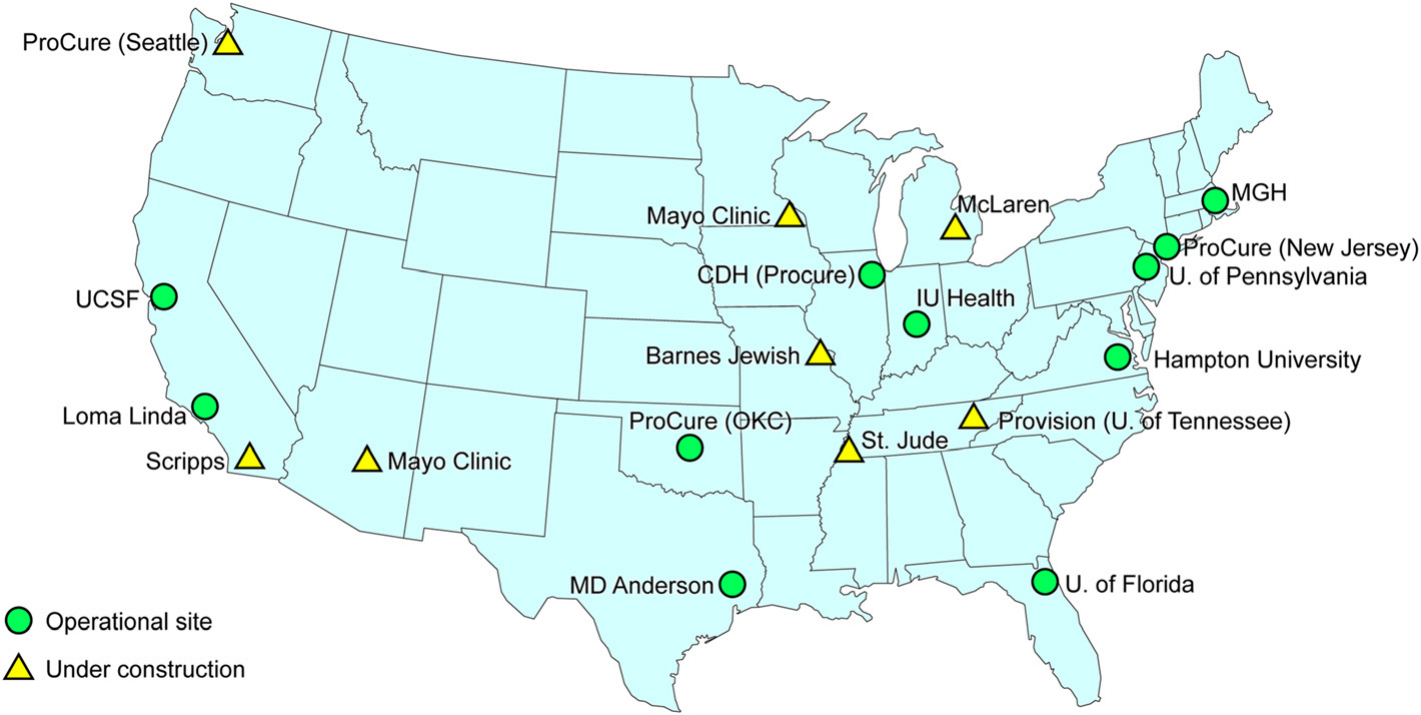
Proton beam treatment facilities that are operational or under construction in the United States. CDH indicates Central Dupage Hospital; IU, Indiana University; MGH, Massachusetts General Hospital; OKC, Oklahoma City; U, University; UCSF, University of California, San Francisco.

**Figure 2:**
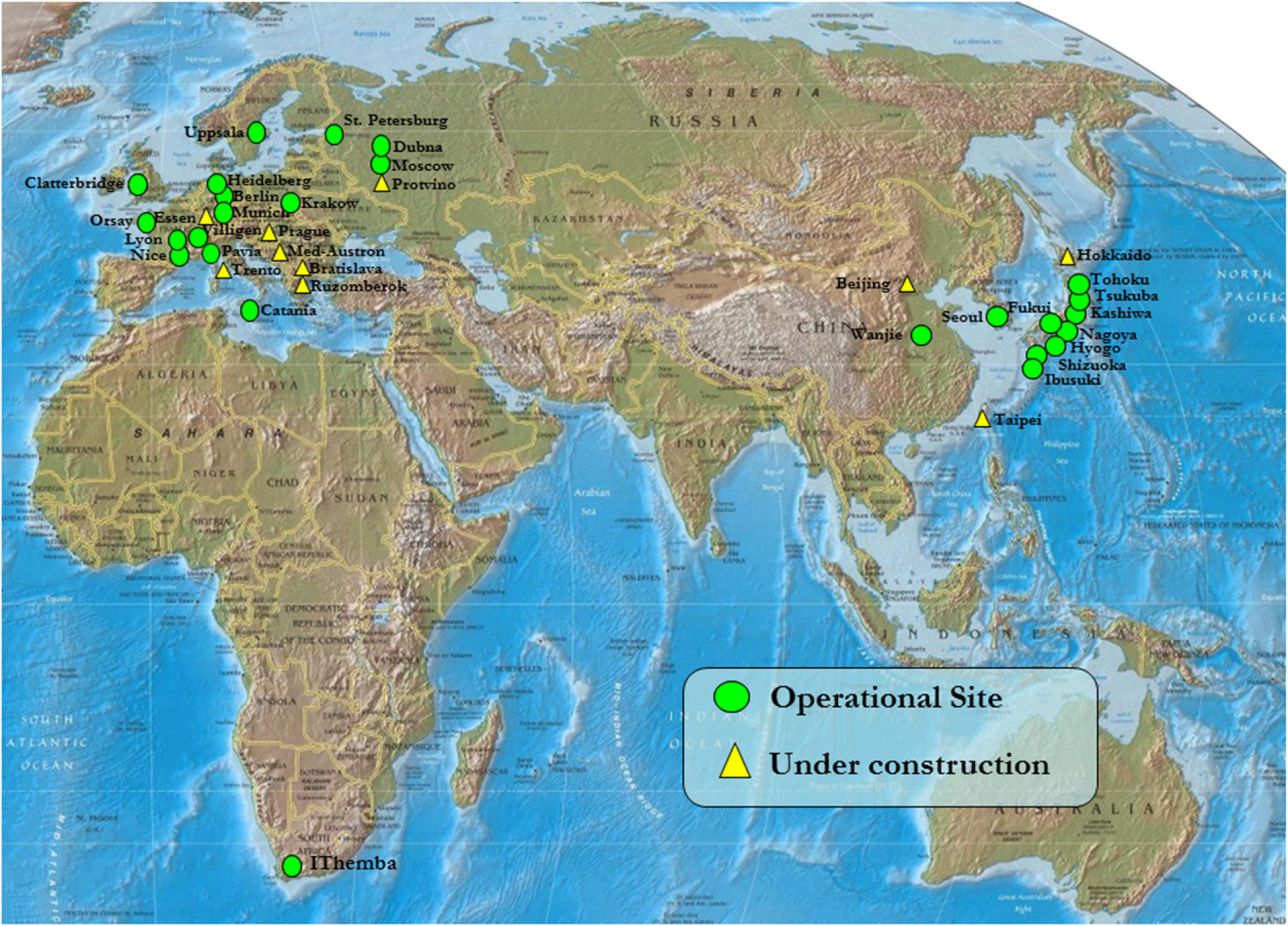
Proton beam treatment facilities that are operational or under construction outside the United States.

Proton beam therapy offers an option for obtaining that balance. Hospital- or clinic-based proton beam facilities have been in existence since 1990. Currently, 11 proton beam facilities are in operation in the United States (Figure 1) and 26 are operational in 13 other countries (Russia, Switzerland, Sweden, England, France, South Africa, Canada, Germany, Japan, Italy, China, South Korea, and Poland) (Figure 2). Eighteen proton beam facilities are under construction in Switzerland, Czech Republic, Austria, Italy, China, Germany, Taiwan, Russia, Slovak Republic, Sweden, and the United States.

Protons are positively charged subatomic particles that are massive compared with x-rays. The biologic effects of protons and x-rays on cells are similar since both are sparsely ionizing with a relatively small linear energy transfer. However, the way protons interact with matter provides advantages compared with x-rays. As protons enter the body, they deposit a very low entrance dose. The depth of proton penetration is dependent on kinetic energy and, hence, the higher the energy, the deeper is the penetration. When the proton arrives at its target, it delivers the dose and stops, thereby eliminating an exit dose. This physical advantage serves to lower the dose to healthy organs both superficial and deep to the tumor, thus reducing the risk of injury. It also allows administration of a higher dose to the tumor, potentially reducing the recurrence rate without increasing the complication rate and leading to better organ function and quality of life. This result can lead to an avoidance of costs associated with treating recurrent tumors and damaged organs. This effect is particularly important in young children with a high likelihood of cure who are strongly susceptible to the long-term effects of x-ray therapy and in patients with cancers located adjacent to critical healthy organs, such as the eye, brain, brainstem, spinal cord, lung, heart, liver, bowel, and kidneys.

### Clinical review

#### Ocular (choroidal) melanoma

Treatment options for patients with ocular melanoma include 1) enucleation, 2) suturing of a radioactive plaque to the eye overlying the melanoma, and 3) proton beam therapy. No differences in survival between treatments have been reported in prospective clinical trials [2,3]. Advantages of the radioactive plaque and proton beam therapy are preservation of the eye and vision. In a phase 3 study performed to compare the radioactive plaque with helium ion therapy (helium ions contain 2 protons), there were fewer recurrences of the melanoma in those patients treated with helium ions (0% vs 13.3%) [3]. The proportion of patients who required enucleation because of melanoma recurrence or complications was less when they were treated with helium ion therapy (9.3% vs 17.3%). The proportion of eyes with visual acuity greater than 20/40 was the same with both treatments (21%-23%).

The Mayo Clinic experience with radioactive plaques has been reported [4]. The recurrence rate is 8%; the enucleation rate is 8%. The proportion of patients with visual acuity greater than 20/40 is 22%. Several large series of patients treated with proton beam therapy have been reported, confirming low recurrence rates (3%-4%) and low enucleation rates (9.4%-11%), with visual acuity greater than 20/40 in 44.8% [5-9]. Of these series, the larger ones included 1,406 patients treated in France [8]; 2,435 patients in Switzerland [9]; and 2,815 patients in Boston, Massachusetts [7]. A phase 3 study conducted by the Massachusetts Eye and Ear Infirmary compared 2 different dose levels of proton beam therapy, 50 Gy and 70 Gy [10]. No differences were noted in outcome, with recurrence rates of 2% to 3% and enucleation rates of 4% to 5%.

Proton beam therapy has a number of advantages over radioactive plaques, including 1) localization requires 1 or no surgery, depending on technique, 2) no hospital stay is needed, yet treatment is still completed in 5 calendar days, 3) more patients are eligible for proton beam therapy than radioactive plaque therapy because of the ability to treat larger tumor sizes and tumors surrounding the optic nerve, and 4) medical staff have no radiation exposure.

#### Skull base chordoma

Skull base chordomas are rare tumors that are difficult to completely remove surgically. Doses of radiation therapy are limited because of the adjacent brain, brainstem, cranial nerves, and spinal cord structures. At Mayo Clinic, with a combination of aggressive surgical debulking, 3-dimensional (3-D) conformal x-ray therapy, and Gamma Knife radiosurgery, the 5-year tumor control rate was 32% in 25 patients [11]. At the Paul Scherrer Institute in Switzerland, with a combination of surgical debulking and scanning proton beam therapy, the 5-year tumor control rate was 81% in 42 patients [12]. The incidence of symptomatic temporal lobe injury at Mayo Clinic was 10% vs 6% with scanning proton beam therapy at the Paul Scherrer Institute [11,12]. At the Harvard Cyclotron Laboratory, 290 patients with skull base chordomas were treated with scattered proton beams [13]. The 5-year tumor control rate was 73%, with an 8% incidence of temporal lobe injury. It appears from these data that proton beam therapy is more effective than x-ray therapy in producing a greater probability of long-term tumor control without increasing the risk of temporal lobe injury.

#### Lung cancer

Standard treatment for locally advanced, inoperable non–small cell lung cancer includes a combination of chemotherapy and x-ray therapy. Median survival is about 17 months, with 50% of the patients having severe toxicity related to treatment (60 Gy of x-ray therapy) [14,15]. Results of phase 3 studies using combined x-ray therapy (60–64 Gy) and chemotherapy report long-term survivors (3–5 years) in 15% to 18% with a 40% to 80% recurrence rate and 48% to 53% of patients having serious or life-threatening toxicity (ie, esophagitis and pneumonitis) [14,15]. The North Central Cancer Treatment Group (including Mayo Clinic) performed a phase 1 dose-escalating trial and found that, by increasing the x-ray therapy dose to 74 Gy, the median overall survival was favorable at 40 months and the recurrence rate was reduced to 15%, but the serious, life-threatening toxicity continued to be high at 54% [16]. The MD Anderson Cancer Center performed a phase 2 dose-escalating trial using proton beam therapy and confirmed that 74 Gy resulted in a favorable median overall survival (29.4 months) and decreased the recurrence rate to 20% [17]. In addition, the incidence of serious toxicity was reduced with proton beam therapy and included dermatitis (11%), esophagitis (11%), and pneumonitis (2%). These results suggest that there is an opportunity to take advantage of dose escalation with proton beam therapy to prolong survival; lower the recurrence rate; decrease the risk of serious, life-threatening toxicity; and intensify chemotherapy. A recently completed phase 3 study comparing a modest dose increase from 60 Gy to 74 Gy of x-ray therapy failed to demonstrate improved survival with 74 Gy [18]. The assumed reason for this lack of benefit was death due to higher doses of x-ray therapy affecting the heart and lungs adversely [19]. This outcome suggests that further dose escalation with x-rays will not be feasible. Massachusetts General Hospital and MD Anderson Cancer Center are nearing completion of a phase 3 clinical trial comparing x-rays to protons in the treatment of locally advanced non–small cell lung cancer.

#### Hodgkin lymphoma in children, adolescents, and young adults

Hodgkin lymphoma (HL) is a curable hematogenous malignancy that affects primarily children and young adults and in which consolidation x-ray therapy is often used after chemotherapy for treatment of initially involved lymph node groups. Survivors of HL have an excessive amount of secondary malignancy (SM), with a 15-year risk of about 15%. Although x-ray therapy may improve outcomes in HL, it may increase the risk of SM, particularly breast, lung, and thyroid cancers and hematogenous cancers. The risk of radiation-induced cancers is proportional to the dose delivered. Using a proton beam treatment planning system, we compared the distribution of proton dose to x-ray dose in patients with HL treated with x-ray therapy at Mayo Clinic. We found that the integral dose of exposure radiation to the patient was reduced by at least 50% with the use of scattered or scanned proton beams compared with 3-D or intensity modulated x-ray beams. This reduction predicts that the risk of radiation-induced cancers would be reduced by at least 50%.

A study from Massachusetts General Hospital of 1,450 patients treated with proton beam therapy at the Harvard Cyclotron Laboratory appears to support this conclusion [20]. A subset of the patients (n=503) was matched to similar patients identified in the Surveillance Epidemiology and End Results cancer registry who were treated with x-ray therapy (n=1,591). SM was reported in 12.8% of the patients treated with x-ray therapy compared with 6.4% in patients treated with proton beam therapy (adjusted hazard ratio, 2.73; 95% confidence interval, 1.87-3.98; *P*<.0001). These data substantiate what we would have predicted by evaluating the x-ray vs proton beam dosimetry. Other investigators have similarly concluded that proton beam therapy may reduce the risk of SM by up to 50% in comparison with x-ray therapy [21].

Similarly, cardiac irradiation during HL x-ray therapy has been associated with an increased risk of coronary artery disease and valvular dysfunction [22,23]. Therefore, a substantial reduction in health care costs, lost productivity, morbidity, death, and human suffering in HL survivors could be realized with the use of proton beam therapy.

#### Esophageal and gastroesophageal junction cancers

The current standard of care for locally advanced esophageal cancer is concurrent chemotherapy and x-ray therapy with or without surgical resection. The 5-year overall survival rate is 20% to 30%. The incidence of treatment-related toxicity that is severe or worse is 33% [24]. Irradiation of the heart has been shown to increase the risk of a myocardial perfusion abnormality [25]. A recent review of the Mayo Clinic experience found that the incidence of non–cancer-related deaths within the first year after treatment was 8% following chemotherapy and x-ray therapy without surgery, 33% following preoperative chemotherapy and x-ray therapy, and 20% following postoperative chemotherapy and radiation therapy. The majority of these deaths were due to cardiopulmonary toxicity. The risk of cardiac toxicity due to x-ray therapy is dependent on the dose delivered and the volume of heart exposed to radiation [1]. Through comparative treatment planning analysis of x-ray and proton beams, we found that the dose delivered to one-third the volume of the heart was reduced from 75% of the dose prescribed to the tumor with conventional x-ray therapy to 58% with intensity modulated x-ray therapy and to just 9% with proton beam therapy. This finding suggests that cardiac-related morbidity and death could potentially be reduced by using proton beam therapy to treat esophageal cancer.

#### Pediatric cancers

Great advances have been made in the treatment of pediatric cancers. Currently, 85% of pediatric cancer patients are cured, although 65% of long-term survivors have chronic health conditions, with death from a SM or other treatment-related event occurring in 20% [26-32]. Despite the increased use of chemotherapy in the management of pediatric cancers, x-ray therapy has an important role in the treatment of approximately 50% of children with cancer, particularly children with brain tumors. However, although x-ray therapy is effective in many children, their quality of life is frequently compromised by late effects of x-ray therapy.

In pediatric practice, the relatively large volume of the body exposed to low doses of x-ray therapy is frequently clinically relevant in relation to long-term effects. Treatment complications include neurocognitive deficits, hearing loss, pituitary dysfunction, hypothyroidism, cardiac dysfunction, pulmonary disease, diminished vertebral body growth, scoliosis, gastrointestinal tract dysfunction, infertility, and SM. The marked reduction in dose to the body and healthy organs associated with proton beam therapy may be used to reduce the extent of the harmful low-dose x-ray effect and thus may be clinically beneficial in pediatric radiation oncology practice.

Medulloblastoma is the second most common pediatric brain tumor. This tumor classically develops in the posterior fossa with frequent metastases along the craniospinal axis, and thus craniospinal axis x-ray therapy is a vital component of treatment. Once considered incur-able, the 5-year overall survival rate is now in excess of 65% following treatment with a combination of surgery, x-ray therapy, and chemotherapy. This rate raises concerns regarding long-term, treatment-associated adverse effects.

Of utmost concern are the neuropsychological effects of x-ray therapy to the central nervous system, including impaired neurocognitive development and behavioral disorders. These effects are dose and volume dependent. There is evidence—from whole-brain irradiation for leukemia—of a dose–response effect on long-term neuropsychological effects [33]. In a recent study of children treated for medulloblastoma, Grill et al. [34] showed a significant correlation between full-scale IQ scores and x-ray dose, with mean scores of 84.5, 76.9, and 63.7 for 0, 25, and 35 Gy, respectively. A dose–response curve relating the probability of neuropsychological sequelae to brain dose has been derived from an analysis of the medical literature [33]. Mulhern et al. [35] prospectively examined the neuropsychological functioning of children with medulloblastoma treated in the POG 8631/CCG 923 study. They found that children treated with 23.4 Gy craniospinal axis x-ray therapy had less neuropsychological toxicity than those treated with 36 Gy.

Long-term effects from x-ray therapy for pediatric cancers include hypoplasia of soft tissue and bone. In children treated with abdominal x-ray therapy for Wilms tumor, 19.6% were reported to have clinically significant long-term orthopedic deficits [36]. There is evidence that the severity of these effects is dose related [37]. Other long-term effects include hearing loss; primary hypothyroidism; thyroid cancer; cardiomyopathy, especially when x-ray therapy is combined with anthracycline chemotherapy; cardiac valvular disease; early onset coronary artery disease; infertility related to pelvic x-ray therapy; and secondary osteosarcoma related to x-ray therapy for Ewing sarcoma, retinoblastoma, or medulloblastoma.

The goal of clinicians is not only to eradicate the primary tumor, but also to minimize the risk of radiation-induced cancers over the lifetime of these children. The observation that many of the late effects of x-ray therapy appear to be dose dependent provides the rationale for proton beam therapy reducing some of the effects that result from exposing structures outside the tumor target volume to radiation.

Comparison of treatment plans using proton, conventional x-ray, or intensity modulated x-ray beams has showed improved dose distributions with proton beams, with modeling estimating a 2-fold reduction or more in risk of a radiation-induced cancer for a child with rhabdomyosarcoma and an 8- to 15-fold decrease for a child with medulloblastoma (due to larger treatment volume) [38]. A study comparing the risk of radiation-induced cancer following spinal irradiation for childhood medulloblastoma after various radiation delivery techniques found the highest lifetime risk of SM with intensity modulated x-ray therapy (30%) and the lowest risk with intensity modulated proton therapy (4%) (Table 1) [39]. These studies underscore the concern with using x-ray therapy in the treatment of pediatric cancers.

**Table 1:** Estimated risk of radiation-induced cancer by radiation delivery technique following spinal irradiation for childhood medulloblastoma

** Radiation delivery technique**	**Risk of radiation-Induced cancer, %**
Intensity modulated x-ray beam	30
Electron beam	21
Conventional x-ray beam	20
Intensity modulated electron beam	15
Intensity modulated proton beam	4

Data from Mu et al. [39].

Table 2 illustrates a comparison of 3 radiation therapy treatment delivery techniques for a child with medulloblastoma [40]. The substantial sparing of healthy tissue is apparent in proton beam therapy of the posterior fossa and spinal axis.

**Table 2:** Dose to cochlea and heart by radiation delivery technique following craniospinal irradiation for childhood medulloblastoma

** Radiation delivery technique**	**Dose to 90% of the cochlea, %**	**Dose to 50% of the heart volume, %**
Conventional x-ray beam	101.2	72.2
Intensity modulated x-ray beam	33.4	29.5
Proton beam	2.4	0.5

Data from St Clair et al. [40].

A recent publication from Sweden projected decreased health care expenses using proton beam therapy in the treatment of pediatric medulloblastoma [41]. The initial cost of proton beam therapy (€10,217.90) was approximately 2.5 times the initial cost of x-ray therapy (€4,239.10). However, the cost of treating adverse events related to x-ray therapy (€33,857.10) was 8 times greater than the cost of treating adverse events related to proton beam therapy (€4,231.80). Considering both initial cost of treatment and the cost of treating adverse events related to the treatment, x-ray therapy was 2.6 times more costly than proton beam therapy (€38,096.20 vs €14,449.70). The additional costs related to treating adverse events associated with x-ray therapy were due to IQ loss, hearing loss, growth hormone deficiency, hypothyroidism, osteoporosis, and SM.

Dosimetric and clinical studies have demonstrated the benefits of proton beam therapy compared with x-ray therapy in reducing dose and harm to healthy organs in children with retinoblastoma, medulloblastoma, pelvic soft tissue sarcoma, bone sarcoma, and orbital rhabdomyosarcoma [42,43].

#### Breast cancer

The meta-analysis of the Early Breast Cancer Trialists’ Collaborative Group demonstrated improved 5-year tumor control and improved 15-year breast cancer and overall mortality rates with the use of adjuvant x-ray therapy [44]. The therapy was used in the clinical setting of breast-conserving and postmastectomy treatment. However, x-ray therapy was associated with an excess of SM (lung cancer relative risk [RR], 1.61 *P*=.0007]; esophageal cancer RR, 2.06 *P*=.05]; leukemia RR, 1.71 *P*=.04]; soft tissue sarcoma RR, 2.34 *P*=.03]; contralateral breast cancer RR, 1.18 *P*=.002]) and non–breast cancer deaths (any non–breast cancer RR, 1.12 *P*=.001]; pulmonary embolism RR, 1.94 *P*=.02]; heart disease RR, 1.27 *P*=.0001]; lung cancer RR, 1.78 *P*=.0004]; and esophageal cancer RR, 2.4 *P*=.04]). The non–breast cancer deaths reduce the efficacy of x-ray therapy by 20%. In patients with positive axillary lymph nodes undergoing postmastectomy chest wall and nodal x-ray therapy, 30% die of noncancer-related deaths. If the morbidity and death due to SM and cardiopulmonary disease could be reduced or eliminated, the overall survival advantage for women treated with x-ray therapy would be further improved, along with reduction in human suffering and health care costs. The risk of cardiotoxicity is increasingly important in light of the cardiotoxicity associated with anthracycline and, more recently, trastuzumab treatment, which are mainstays in modern adjuvant medical therapy for breast cancer.

We developed 3 treatment plans for a cohort of women with left-sided stage I breast cancer who were undergoing breast-conserving therapy at Mayo Clinic with lumpectomy and breast irradiation. The first plan used conventional x-ray therapy to the entire breast, with an electron boost to the tumor cavity; the second used passively scattered proton beams; and the third used actively scanned proton beams. Compared with the x-ray and electron boost plan, the 2 proton beam plans substantially reduced all measures of lung dose. For example, the mean total lung dose was reduced by 71% using the passively scattered beams and by 81% using the actively scanned beams. Both proton beam plans eliminated the dose to the contralateral lung. The 2 proton beam plans also reduced all measures of dose to the heart. For example, the mean total heart dose was reduced by 75% with the passively scattered beams and by 99% with the actively scanned beams. The mean dose to the contralateral breast was reduced with the proton beam plans compared with the x-ray and electron beam plan—by 88% using the passively scattered beams and by 96% using the actively scanned beams. In addition, the 2 proton beam plans reduced the mean dose to the entire body by 37% for the passively scattered proton beams and by 54% for the actively scanned proton beams.

A similar dosimetric study of women undergoing postmastectomy chest wall and regional lymph node irradiation at Mayo Clinic revealed similar advantages to protons compared with x-rays. Other investigators have confirmed these findings [45-49]. Lundkvist et al. [50] have demonstrated that proton beam therapy is cost-effective in women with left-sided breast cancer and risk factors for cardiac disease, on the basis of a lower cost per quality-adjusted life-year. Cost-effectiveness will be further improved when investigators also include the cost reductions associated with a reduced incidence of radiation-induced malignancy and pulmonary disease. It is hypothesized that proton beam therapy will be most cost-effective in young women with left-sided breast cancer; in women with a long life expectancy; and in women with risk factors for cardiopulmonary disease, a desire to avoid mastectomy, and indications for postmastectomy chest wall and nodal irradiation.

Current clinical trials are evaluating the safety, efficacy, and cosmetic outcome of partial breast irradiation compared with whole breast irradiation. Should these trials document a meaningful clinical advantage to partial breast irradiation, Taghian et al. [48], Kozak et al. [49,51], and Bush et al. [52,53] have demonstrated that partial breast irradiation using proton beam therapy is safe, effective, and technically feasible; provides excellent tumor coverage; and improves healthy tissue (heart and lung) sparing, including nontarget breast tissue, when compared with partial breast irradiation using conventional x-rays and electron beams. In addition, it is less expensive than intracavitary and interstitial brachytherapy.

#### Prostate cancer

The treatment of prostate cancer with proton beam therapy is controversial. Current treatment options include prostatectomy, brachytherapy, and intensity modulated x-ray therapy, all of which are less costly than proton beam therapy.

Prospectively randomized clinical trials have demonstrated that higher doses of x-ray therapy result in improved survival and lower doses to the rectum and bladder result in lower risks of complications. A phase 3 study funded by the NHS Trust randomly assigned 225 men with prostate cancer to 2-dimensional (2-D) or 3-D conformal x-ray therapy [54]. The results of this study proved the principle that reducing the radiation dose to the rectum and bladder by using 3-D conformal techniques reduces the incidence of ≥grade 2 bowel toxicity (from 18% with 2-D to 8% with 3-D) and bladder toxicity (from 23% with 2-D to 20% with 3-D). A phase 3 dose-escalation study conducted by MD Anderson Cancer Center randomly assigned 301 patients to 70 Gy using 2-D x-ray therapy vs 78 Gy using 3-D conformal x-ray therapy [55,56]. This study proved that a higher dose of x-ray therapy results in fewer recurrences (79% 5-year freedom from biochemical failure with 78 Gy vs 69% with 70 Gy). The ≥grade 2 bowel and bladder toxicity was 14% (bowel) and 20% (bladder) for 2-D compared with 21% (bowel) and 9% (bladder) for 3-D x-rays.

The reduced recurrence rate using a higher dose has been confirmed by another phase 3 clinical trial conducted by the Netherlands Cancer Institute, in which 664 men were randomly assigned to 68 Gy vs 78 Gy using 3-D conformal x-ray therapy [57]. The 5-year freedom from biochemical failure was increased from 53% with 68 Gy to 66% with 78 Gy. The ≥grade 2 bowel toxicity was 27% with 68 Gy and 32% with 78 Gy; the ≥grade 2 bladder toxicity was 41% with 68 Gy and 39% with 78 Gy. Intensity modulated x-ray therapy has been administered to a dose as high as 81 Gy in 561 men with prostate cancer at Memorial Sloan-Kettering Cancer Center, with ≥grade 2 bowel toxicity of just 1.6% and ≥grade 2 bladder toxicity of 12% [58]. Loma Linda University Medical Center has reported the results of treating 1,255 men with prostate cancer to 74 Gy using proton beam therapy [59]. The 5-year freedom from biochemical failure was 75%, with 3.5% ≥grade 2 bowel toxicity and 5.4% ≥grade 2 bladder toxicity.

Finally, Massachusetts General Hospital and Loma Linda University Medical Center conducted a phase 3 dose-escalation study in 393 patients with prostate cancer using a combination of x-rays with a proton beam boost to the prostate gland [60]. Patients were randomly assigned to 70.2 Gy or 79.2 Gy. There was a significant improvement in the 5-year freedom from biochemical failure rate in men randomly assigned to 79.2 Gy (80%) compared with those randomly assigned to 70.2 Gy (61%) (*P*<.0001). This improvement in recurrence rate with higher dose was obtained without significantly increasing the risk of ≥grade 2 bowel toxicity (9% with 70.2 Gy vs 18% with 79.2 Gy) or bladder toxicity (20% with 70.2 Gy vs 21% with 79.2 Gy). Only 2% of patients in both treatment arms had late severe (≥grade 3) genitourinary toxicity and 1% of patients in the high-dose arm had late ≥grade 3 gastrointestinal tract toxicity [61]. A cost-effectiveness study suggested that proton beam therapy may be cost-effective in young men with intermediate-risk prostate cancer who have longer life expectancy [62].

In summary, direct evidence shows that the higher the dose of radiation administered, the lower the risk of prostate cancer recurrence. Direct evidence also shows that lower doses to the rectum and bladder are associated with a lower risk of complications. Indirect evidence shows that with highly conformal techniques (intensity modulated x-ray therapy or proton beam therapy), the dose can be further escalated without increasing the risk of bowel or bladder toxicity and, in fact, with a lower risk of harm than with conventional 2-D and 3-D x-ray therapies. A phase 3 study comparing high-dose intensity modulated x-ray therapy with proton beam therapy was recently opened to patient accrual.

One way of drastically reducing the cost of proton beam therapy compared with intensity modulated x-ray therapy and bringing it more in line with prostatectomy and brachytherapy is to reduce the number of treatments from 40 to 45 administered over 8 or 9 weeks to 5 treatments administered in 1 week [63]. This regimen would be far more convenient for patients and reduce their time off work and their out-of-pocket expenses. Clinical trials evaluating the safety and efficacy of hypofractionated proton beam therapy should be designed and conducted.

#### Head and neck cancer

Evidence exists that patients with head and neck cancer may benefit from proton beam therapy by increasing the dose to the cancer to reduce recurrence risk and by reducing the dose to the salivary glands, mandible, and maxilla to lower the risk for dry mouth, dental caries, dental extractions, and osteoradionecrosis [64-67]. The risk of osteoradionecrosis has been shown to be associated with the total dose and the dose per treatment received by the mandible, with a 0% risk for less than 54 Gy at 1.8 Gy per treatment and a 9.8% risk for 54 Gy or greater at 1.8 Gy per treatment [68]. We evaluated a cohort of consecutive patients at Mayo Clinic undergoing postoperative adjuvant x-ray therapy for tongue cancer. We planned that each patient would receive intensity modulated x-rays and actively scanned proton beams. All measures of mandibular dose were significantly reduced in the patients receiving actively scanned proton beams. For example, the mean volume of the mandible receiving 54 Gy with intensity modulated x-ray beams was 61% (range, 37%-90%) compared with 26% (range, 6%-51%) with actively scanned proton beams (*P*=.002). Furthermore, the mean parotid dose was reduced from 33.1 Gy (range, 24.2-44.1 Gy) with intensity modulated x-ray beams to 19.3 Gy (range, 9.6-32.6 Gy) with actively scanned proton beams (*P*=.002), thereby significantly reducing the risk of xerostomia [69].

#### Other cancers

Potential advantages of proton beam therapy exist for rectal and anal cancers (lower dose to bowel, bladder, and hips); gastric, pancreatic, and hepatobiliary cancers (lower dose to liver, small bowel, heart, lungs, kidneys, and spinal cord); and bone and soft tissue sarcomas. Improved hematologic tolerance may allow dose intensification of chemotherapy given concurrently with proton beam therapy for thoracic, gastrointestinal tract, and other cancers [17].

### Take home points

Mayo Clinic is a national provider of health care with clinics and hospitals in Arizona, Florida, Georgia, Iowa, Minnesota, and Wisconsin caring for more than 13,000 new cancer patients annually. The most common types of cancer treated with radiation therapy at Mayo Clinic are breast cancer (15%), lung cancer (12%), prostate cancer (11%), gastrointestinal tract cancers (10%), and head and neck cancer (5%). In 2002, the Department of Radiation Oncology participated in a departmental exercise to review its status and determine its future direction. As a result of this exercise, the implementation of a charged particle therapy program became a top priority.

There is always a dilemma for large medical practices on the timing for the implementation of new technologies. *Health technology assessment* is the systematic evaluation of properties, effects, or other impacts of health technology. The main purpose of a health technology assessment is to inform decision making for policy decisions related to technology in health care. The assessment may address the direct and intended consequences of technologies, as well as their indirect and unintended consequences. Historically, the emphasis has been on technology assessment among hospitals, health systems, and health plans. The most common form of technology assessment has focused around pharmaceuticals through pharmacy and therapeutics committees [70]. However, in recent years the interest has been increasing in technology assessment around devices and procedures. For example, 64-slice computed tomography, positron emission tomography, da Vinci robots, health information technology systems, and telemonitoring programs have undergone technology assessments commonly. Typically, the goal of these committees is to weigh the benefits and costs and conduct analyses of return on investment of new technologies. This evaluation also allows health systems to identify priorities for investment.

We recognize the need to generate, evaluate, integrate, and manage knowledge and information related to proton beam therapy. To transform the cancer care delivery process and to be trusted and affordable through the reduction of harm and cost to patients and society, health care providers will need to define which patients benefit the most from proton beam therapy and to define outcomes (tumor control, overall survival, patient-reported function and quality of life, and cost-effectiveness) prospectively in controlled clinical trials and registries.

## Abbreviations

HL: Hodgkin lymphoma; RR: Relative risk; SM: Secondary malignancy; 3-D: 3-dimensional; 2-D: 2-dimensional.

## Competing interests

The authors declare that they have no competing interests.

## Authors' contributions

All of the authors participated in the writing, reviewing, and editing of the manuscript. All authors read and approved the final manuscript.
